# Dasatinib and Quercetin Limit Gingival Senescence, Inflammation, and Bone Loss

**DOI:** 10.1177/00220345241299789

**Published:** 2025-01-10

**Authors:** K. Rattanaprukskul, X.-J. Xia, M. Hysa, M. Jiang, M. Hung, S.F. Suslavich, S.E. Sahingur

**Affiliations:** 1 Department of Periodontics, School of Dental Medicine, University of Pennsylvania, Philadelphia, PA, USA; 2 Department of Periodontology, Faculty of Dentistry, Chulalongkorn University, Bangkok, Thailand

**Keywords:** periodontal, senotherapy, aging, metabolism, senescence associated phenotype (SASP), senescent cell

## Abstract

Cellular senescence has emerged as one of the central hallmarks of aging and drivers of chronic comorbidities, including periodontal diseases. Senescence can also occur in younger tissues and instigate metabolic alterations and dysfunction, culminating in accelerated aging and pathological consequences. Senotherapeutics, such as the combination of dasatinib and quercetin (DQ), are being increasingly used to improve the clinical outcomes of chronic disorders and promote a healthy life span through the reduction of senescent cell burden and senescence-associated secretory phenotype (SASP). Recent evidence suggests that senescent cells and SASP can contribute to the pathogenesis of periodontal diseases as well. In this study, we investigated the effect of DQ interventions on periodontal tissue health using preclinical models of aging. In vitro, DQ ameliorated biological signatures of senescence in human gingival keratinocytes upon persistent exposure to periodontal bacteria, *Fusobacterium nucleatum*, by modulating the levels of key senescence markers such as p16, SA-β-galactosidase, and lamin-B1 and inflammatory mediators associated with SASP including interleukin-8, matrix metalloproteinase (MMP)–1, and MMP-3. In vivo, the oral administration of DQ mitigated senescent cell burden and SASP in gingival tissues and reduced naturally progressing periodontal bone loss in aged mice. Collectively, our findings provide proof-of-concept evidence for translational studies and reveal that targeting gingival senescence and the senescence-associated secretome can be an effective strategy to improve periodontal health, particularly in vulnerable populations.

## Introduction

The prevalence of periodontal diseases reaches more than 70% among those 65 y and older (Eke et al. 2020). Cellular senescence is the central hallmark of aging and driver of several chronic diseases such as diabetes, cardiovascular disease, kidney disease, Alzheimer’s disease, and osteoporosis (Chaib et al. 2022; Farr et al. 2024). Senescence is a response to chronic stressors including physical, chemical, or biological factors such as persistent inflammation, oxidative stress, and dysbiotic microbiome. Senescent cells can instigate metabolic changes and organ dysfunction, perpetuating disease initiation and/or progression and culminating in accelerated aging (Zhu et al. 2024). Characteristics of senescent cells include cell-cycle arrest, morphological changes, defective nuclear membrane, and metabolic alterations (González-Gualda et al. 2021; Chaib et al. 2022). They remain metabolically active and secrete a complex set of proinflammatory cytokines, chemokines, and matrix metalloproteases, known as the senescence-associated secretory phenotype (SASP) (Huang et al. 2022). Senescent cells can also interfere with the function of surrounding cells, fostering further chronic inflammation termed *inflammaging* (Huang et al. 2022). The cumulative detrimental effects of senescent cells, which include reduced replicative capacity and persistent inflammatory secretome, can disrupt tissue homeostasis and worsen periodontal damage, affecting both younger and, to a greater extent, aging tissue (Albuquerque-Souza et al. 2022; Albuquerque-Souza et al. 2024; Guo et al. 2024; Li et al. 2024; Rattanaprukskul et al. 2024). Therefore, similar to other chronic conditions, targeting senescence in the oral mucosa can be a novel strategy to maintain health (Zhu et al. 2024).

Senotherapeutics are a new class of drugs that selectively target senescent cells and the senescence-associated secretome (Zhu et al. 2015; Chaib et al. 2022). The combination of (D) and quercetin (Q) is regarded as one of the most promising and safe senotherapeutics (Gonzales et al. 2023). D is a tyrosine kinase inhibitor and Food and Drug Administration–approved agent for leukemia (Zhu et al. 2015). Q is a flavonoid extracted from plants with potent antioxidant, anti-inflammatory, and antiaging properties through its effect on a wide range of biochemical pathways, including NF-κB, PI3K, and Bcl cascades (Mooney et al. 2021; Zhang et al. 2023). Both substances synergistically reduce senescent cell burden and senescence-associated secretome (Zhu et al. 2015; Zhang et al. 2023). Preclinical studies in animals have demonstrated the reduction of senescent cells and the improvement of physiological functions in conditions that share similar pathophysiology with periodontal diseases such as arthritis, obesity, osteoporosis, diabetes, and pulmonary fibrosis after DQ supplementation (Chaib et al. 2022). Clinically, DQ therapy has been shown to improve clinical symptoms in patients with idiopathic pulmonary fibrosis (Nambiar et al. 2023). There are also multiple ongoing clinical trials exploring the benefits of DQ therapy in other conditions, such as chronic kidney disease and Alzheimer’s disease, as well as increasing life span (Chaib et al. 2022; Garbarino et al. 2024). In fact, Q alone has been reported to alleviate gingival inflammation and reduce periodontal bone loss (Mooney et al. 2021; Laky et al. 2024).

In this study, we examined the effect of DQ on gingival tissues by monitoring the levels of selected markers of senescence (p16, SA-β-galactosidase, lipofuscin, and lamin-B1) and inflammatory mediators related to SASP (interleukin [IL]-8, matrix metalloproteinase [MMP]–1, and MMP-3) using established in vitro and in vivo models.

*Fusobacterium nucleatum* is a ubiquitous oral opportunistic bacterium that plays a significant role as a prominent pathogen in numerous disorders distant from the oral cavity including those associated with aging (Brennan and Garrett 2019; Albuquerque-Souza et al. 2024). *F. nucleatum* can trigger senescence-like cellular and functional alterations in gingival keratinocytes through modulation of lysosomal metabolism, cellular proliferation, and nuclear membrane integrity (Albuquerque-Souza et al. 2024). Using this in vitro model, we showed that DQ treatment can alleviate senescence features and secretion of SASP mediators in gingival keratinocytes. Similarly, in vivo, orally administered DQ reduced senescent cell burden, SASP, and naturally occurring alveolar bone loss in older mice compared with vehicle-treated controls. Together, our data indicate that customized senotherapeutic regimens can be a novel strategy to maintain periodontal health via alleviation of senescence phenotype and senescence-associated secretome.

## Materials and Methods

### In Vitro Assays

#### Cell culture

Telomerase immortalized gingival keratinocytes (TIGKs) (ATCC, #CRL-3397) were cultured in DermaLife medium with supplements (rh-insulin, L-glutamine, epinephrine, Apo-transferrin, rh-transforming growth factor–α, extract P, hydrocortisone; LifeLine Cell technology) as described (Albuquerque-Souza et al. 2024). TIGKs were seeded at 6 × 10^5^ cells/well in a 6-well plate until they reached a confluence of 75% and then pretreated with D (10 nM), Q (1 µM), DQ, and vehicle for 2 h followed by challenge with heat-killed *F. nucleatum* at a multiplicity of infection of 1:10 or sham for 6 d. Culture media including therapeutics and bacteria were renewed every 2 d (Li et al. 2020; Albuquerque-Souza et al. 2024).

#### Bacterial culture

*F. nucleatum* (ATCC 25586) was grown under anerobic conditions (90% N_2_, 5% CO_2_, and 5% H_2_, 37 °C) in 3.7% brain heart infusion (Difco Laboratories) enriched with 0.05% extract yeast, 5 µg/mL hemin, 0.5 µg/mL vitamin K, 1 µg/mL N-acetyl muramic acid, 0.1% cysteine (Sigma-Aldrich), and 5% fetal bovine serum. After growth, the bacteria were harvested by centrifugation and resuspended in phosphate-buffered saline. The bacteria were heat killed at 80 °C for 15 min and stored at −80 °C until use (Albuquerque-Souza et al. 2024).

#### Cell viability assay

Cell viability was determined using the Cell Counting Kit-8 (APExBIO) assay. TIGKs were seeded into 96-well plates at 5 × 10^3^ cells/well and cultured overnight. Details are in the appendix.

#### RNA isolation and RT-qPCR

Total RNA was extracted using the RNeasy Plus Mini kit (Qiagen), and reverse transcription quantitative polymerase chain reaction (RT-qPCR) was performed using specific primers (Appendix Table 1) for SYBR Green Master Mix (SABiosciences) in the StepOne PlusSystem (Applied Biosystems) following established protocols (Mooney et al. 2021). Details are in the appendix.

#### Western blot

TIGKs were harvested on day 6, and Western blot was performed as described (Li et al. 2020). Protein levels for p16 and lamin-B1 were assessed. The intensity of the signal obtained for each protein was quantified by densitometry using Alpha View software, and protein expression was calculated using GAPDH as endogenous control. Details are in the appendix.

#### Immunofluorescence assay

TIGKs were harvested on day 6, and immunofluorescence staining was performed as described previously (Albuquerque-Souza et al. 2024). The immunofluorescence was stained for p16 and lamin-B1. Images were taken using a confocal Nikon laser microscope (Nikon Instruments) at 630× magnification or Leica DM6B microscope (Leica) at 40× magnification. Details are in the appendix.

#### SA-β-galactosidase staining

SA-β-galactosidase staining was performed on the cells harvested on day 6 with SA-β-galactosidase staining kit (Cell Signaling Technology #9860) (Albuquerque-Souza et al. 2024). Images were obtained using a digital inverted-phase microscope (Olympus CK40) at 20× magnification. After staining, TIGKs were lysed using RIPA buffer (Sigma-Aldrich), and SA-β-galactosidase activity was measured using the microplate reader (Thermo Fisher Scientific Multiskan) at an optical density (OD) of 600 nm (Ewald et al. 2009; Albuquerque-Souza et al. 2024).

#### Enzyme-linked immunosorbent assay

To carry out the SASP factor analyses, TIGKs were first challenged with heat-killed *F. nucleatum* for 6 d to induce senescence followed by 2 more days of culture in new media including either DQ or vehicle without bacteria. The levels of IL-8, MMP-1, and MMP-3 were detected in cell-free culture supernatants collected on day 6 and day 8 using specific enzyme-linked immunosorbent assay kits (Invitrogen Thermo Fisher Scientific, #88-8086, EHMMP1, and BMS2014-3) (Mooney et al. 2021).

### In Vivo Studies

#### Animals

All animal procedures were approved by the Institutional Animal Care and Use Committee at University of Pennsylvania (Protocol No. 806809) and followed ARRIVE 2.0 guidelines (appendix). BALB/c mice (Jackson Laboratory) were maintained in a specific pathogen-free environment and housed in a temperature-controlled, air-conditioned room on a 12-h light-dark cycle. To evaluate the effect of DQ on periodontal health, we used a naturally occurring bone loss model in aged mice. Nine 15- to 16-mo-old mice (2 males and 7 females) were orally administered D (5 mg/kg) and Q (50 mg/kg) in 10% PEG400 for 3 consecutive days every 2 wk, while 8 mice (2 males and 6 females) received the vehicle as control for 3 mo (Appendix Fig. 2) (Ruggiero et al. 2023). At the conclusion, the mice were euthanized, gingival tissues were harvested for histological and molecular analyses, and the jaws were collected to determine alveolar bone levels.

#### Histology of mouse gingival tissues

Gingival tissues were stained for SA-β-galactosidase, lipofuscin, and p16 as described previously (Georgakopoulou et al. 2013; Rattanaprukskul et al. 2024). The SA-β-galactosidase–positive areas were quantified using ImageJ Fiji software. The percentage of positive areas was calculated by dividing the stained area by the total tissue area and multiplying by 100 (Crowe and Yue 2019; Jannone et al. 2020). Lipofuscin-positive cells were manually quantified in each field at 40× magnification, and a minimum of 3 randomly selected regions/fields were evaluated for quantification. The relative fluorescence intensity of p16 was calculated using ImageJ Fiji software. Details are in the appendix.

#### Determination of mRNA expressions of senescence markers and inflammatory mediators related to SASP

Total RNA isolation and RT-qPCR were performed on the gingival tissues following established protocols using specific primers (Appendix Table 2) (Mooney et al. 2021).

#### Alveolar bone loss assessments

Alveolar bone levels were determined using a Nikon microscope (Nikon Instrument Inc., SMZ800) with a 40× objective and NIS-Elements software (Nikon Instrument Inc.) following established protocols (Kim et al. 2015). Details are in the appendix.

### Statistical Analysis

Statistical analyses were conducted using GraphPad Prism Software Inc. The normality distribution of the data was assessed using the Shapiro-Wilk test with Lilliefors correction. For comparisons between the 2 groups, either an unpaired *t* test or Mann-Whitney *U* test was employed. Multiple comparisons were evaluated using either 1-way analysis of variance with Tukey’s post hoc test or Kruskal-Wallis with post hoc Dunn’s test. A significance level of *P* < 0.05 was considered statistically significant.

## Results

### DQ Alleviates Signs of Senescence in Gingival Keratinocytes

We tested the impact of DQ on senescence response in human gingival keratinocytes challenged by prolonged exposure to *F. nucleatum* by monitoring widely used biological signatures of senescence including SA-β-galactosidase, p16, and lamin-B1. To establish the optimal concentration, we first assessed cell viability at different concentrations of the drug regimen and noted 10 nM of D and 1 µM of Q as the effective and safe dosages without any sign of cellular toxicity (Appendix Fig. 3). SA-β-galactosidase is a commonly used indicator of lysosomal and metabolic alterations in senescent cells and recommended as the primary marker for assessing senescence in combination with other selected markers and SASPs (Gil 2023; Zhu et al. 2024).

**Figure 1.**
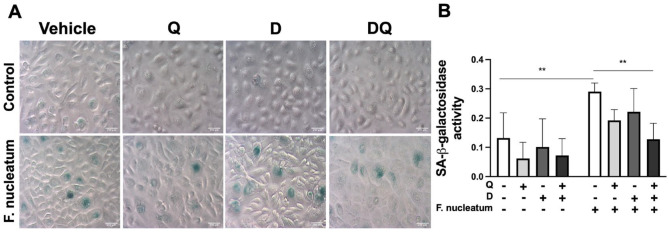
Dasatinib and quercetin (DQ) reduces SA-β-galactosidase in gingival keratinocytes. (**A**) Representative images of SA-β-galactosidase–positive cells (in green) at 20× magnification. (**B**) Quantification of SA-β-galactosidase activity using an optical density of 600 nm. **P ≤ 0.01.

Our results revealed a significant reduction in SA-β-galactosidase activity in gingival keratinocytes, which received Q individually or in combination with D compared with the cells treated with vehicle (Fig. 1A, B). The decrease was more robust in the cells receiving the combined intervention, suggesting that DQ can improve cellular metabolism in gingival keratinocytes.

**Figure 2.**
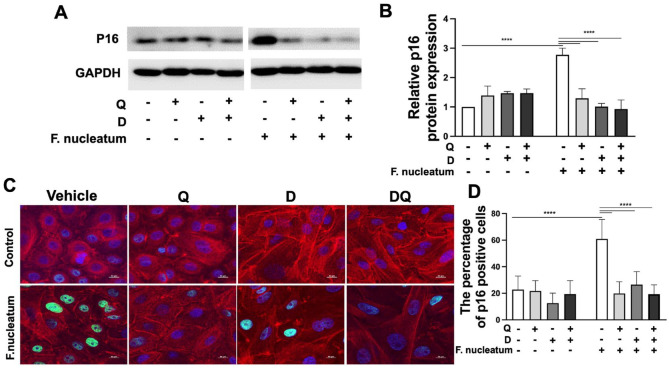
Dasatinib and quercetin (DQ) reduces the senescence marker p16 in gingival keratinocytes. (**A**) Representative Western blots of p16 and (**B**) quantitative analysis. (**C**) Representative immunofluorescence images of p16-positive cells and (**D**) quantitative analysis. ****P ≤ 0.0001.

P16 ^INK4^ is a cyclin-dependent kinase (CDK) inhibitor in the p16/Rb pathway that inhibits the CDK4-CyclinD complex and prevents Rb phosphorylation and thus blocks G1-S progression, leading to the arrest of cell proliferation and growth (Chaib et al. 2022). Further substantiating the impact of this drug regimen on gingival senescence, we were able to show decreased p16 levels using both Western blot (Fig. 2A, B) and immunofluorescence microscopy (Fig. 2C, D) in gingival keratinocytes following interventions with individual agents or combination therapy compared with vehicle controls. These results indicate that DQ can alleviate cell-cycle arrest, a prominent feature of senescence, in gingival cells.

**Figure 3.**
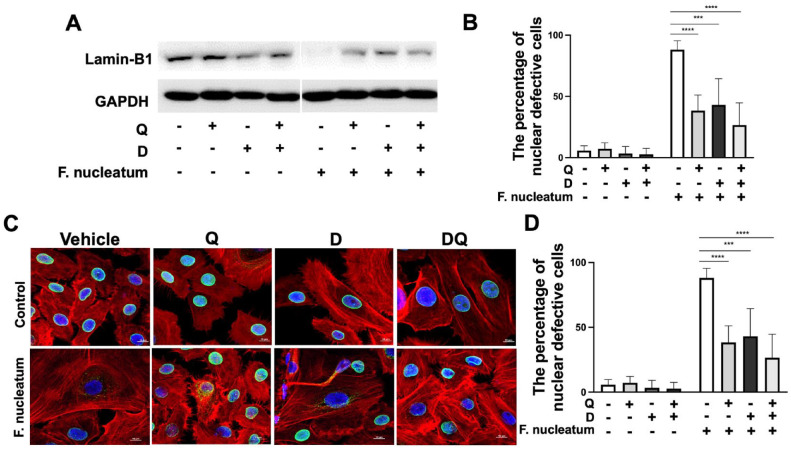
Dasatinib and quercetin (DQ) attenuates nuclear envelope damage in gingival keratinocytes. (**A**) Representative Western blots of lamin-B1. (**B**) Relative protein levels of lamin-B1. (**C**) Representative immunofluorescence images of lamin-B1. (**D**) The percentage of cells exhibiting nuclear defects. ***P ≤ 0.001, ****P ≤ 0.0001.

Another hallmark of a senescent cell is the disintegration of the nuclear envelope, which can be monitored through cellular lamin-B1, a critical protein functioning in the preservation of nucleus integrity. Reduced lamin-B1 expression triggers destabilization and eventual breakdown of the nuclear membrane in senescent cells (González-Gualda et al. 2021). Following treatment, the breakdown of lamin-B1 (Fig. 3A, B) and the percentage of cells displaying nuclear defects (Fig. 3C, D) were significantly decreased, further supporting the impact of senotherapy on gingival cells.

### DQ Reduces Inflammatory Mediators Associated with SASP in Gingival Keratinocytes

**Figure 4.**
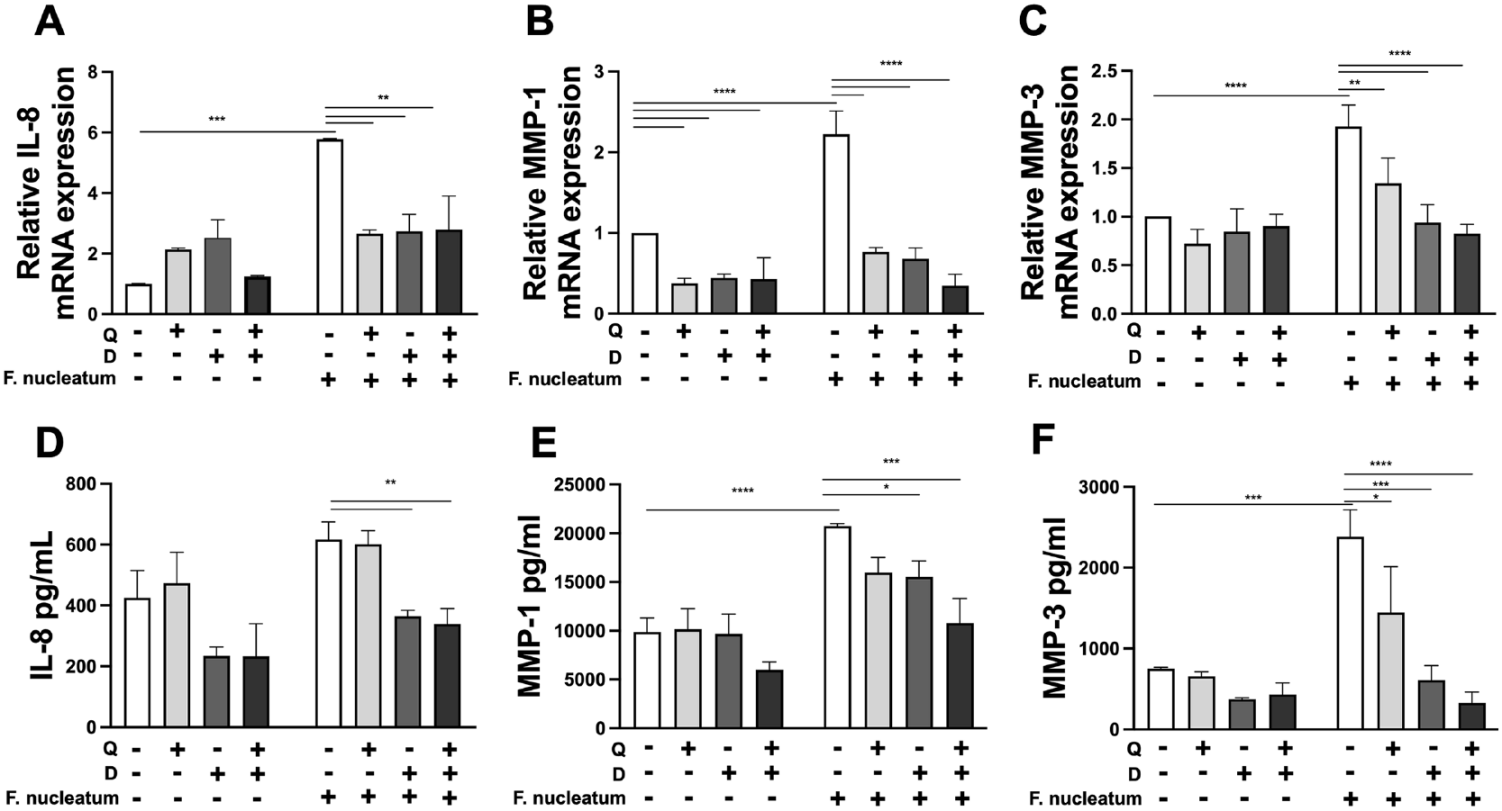
Dasatinib and quercetin (DQ) mitigates inflammatory mediators related to senescence secretome in gingival keratinocytes. (**A–C**) Relative mRNA expressions of interleukin (IL)–8, matrix metalloproteinase (MMP)–1, and MMP-3. (**D–F**) IL-8, MMP-1, and MMP-3 protein levels in culture supernatants were measured with enzyme-linked immunosorbent assay. *P ≤ 0.05, **P ≤ 0.01, ***P ≤ 0.001, ****P ≤ 0.0001.

Senescent cells continue to possess the ability to produce a repertoire of proinflammatory cytokines and proteinases known as SASP, which are also implicated in the pathogenesis of periodontal diseases. These factors can also adversely affect neighboring cells in a paracrine manner, leading to impaired cellular and metabolic function. As a consequence, SASP plays a pivotal role in promoting and propagating the senescence effect, thereby contributing to disease progression and chronicity. DQ treatment reduced IL-8, MMP-1, and MMP-3 gene expression (Fig. 4A–C) and protein levels (Fig. 4D–F) in gingival keratinocytes that are exposed to persistent microbial challenge, providing further evidence for the benefits of these agents for oral health.

### DQ Alleviates Senescence Features in Gingival Tissues and Alveolar Bone Loss in Aged Mice

**Figure 5.**
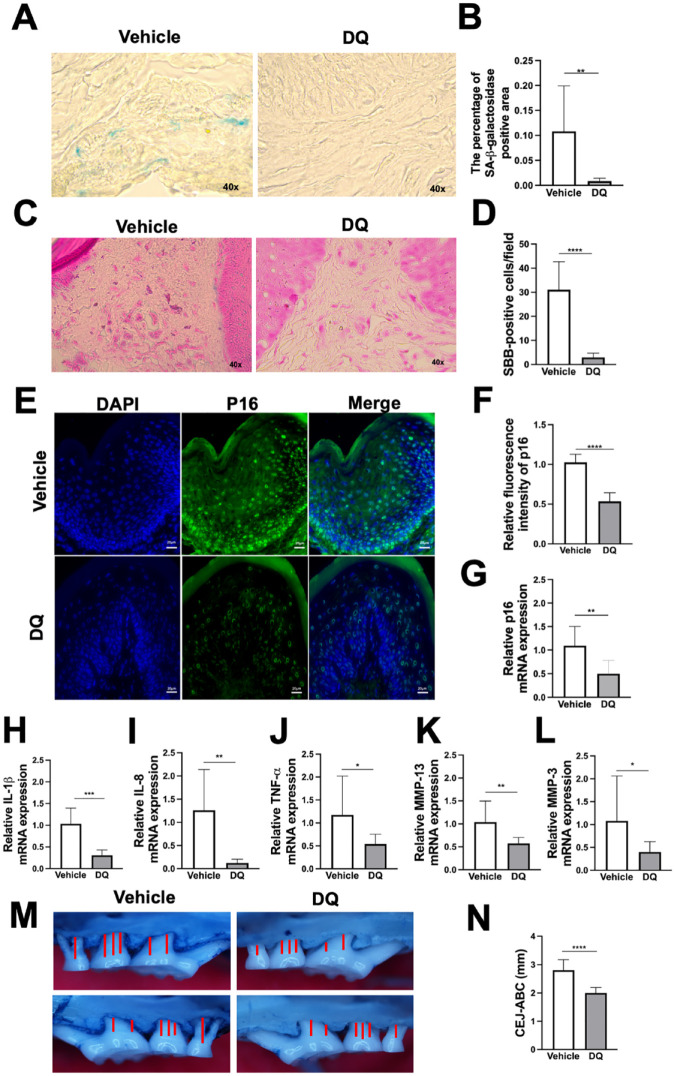
Dasatinib and quercetin (DQ) mitigates gingival senescence and age-related periodontal bone loss. Balb/c mice (15 to 16 mo old) were treated with vehicle (*n* = 9) and DQ (*n* = 8) for 3 consecutive days per week every 2 wk for 3 mo. SA-β-galactosidase (**A, B**), lipofuscin deposition (**C, D**), p16 (**E–G**), senescence-related secretome (**H–L**), and alveolar bone loss (**M, N**). *P ≤ 0.05, **P ≤ 0.01, ***P ≤ 0.001, ****P ≤ 0.0001.

Translation of findings into clinical practice requires testing the effects of novel interventions in established animal models. Aging mouse exhibits naturally occurring alveolar bone loss, which becomes evident at 9 mo and significantly increases after 15 mo of age (Liang et al. 2010). This temporal threshold aligns with the human middle-age phase, during which the prevalence of periodontitis tends to escalate (Eke et al. 2020). We conducted DQ interventions in aging mice for 3 mo and noted significantly decreased SA-β-galactosidase (Fig. 5A, B), lipofuscin (Fig. 5C, D), and p16 (Fig. 5E–G) as well as inflammatory mediators related to senescence secretome, including *Il-1β*, *Il-8*, *Mmp-3*, and *Mmp-13* (*MMP-1* in human) in the gingival tissues of older mice treated with DQ compared with those in the vehicle-treated group (Fig. 5H–L). Notably, younger healthy gingiva did not exhibit signs of senescence, and the DQ intervention successfully reduced the levels of these markers in aged tissues to match those of younger tissues (Appendix Fig. 4). Furthermore, although we observed some variations between genders, the results were inconclusive due to the limited sample size, warranting further studies (Appendix Table 3). Consistently, alveolar bone levels were improved in the mice given DQ compared with the vehicle group at the completion of the experimental period, further highlighting the benefit of this regimen on periodontal health (Fig. 5M, N).

## Discussion

Conventional periodontal therapy faces challenges in effectively managing persistent clinical manifestations. As a result, ongoing research efforts are directed toward developing alternative or complementary strategies to maintain periodontal health, particularly in susceptible populations who typically experience more severe forms of the disease. Continued exposure to various stressors can create an oral tissue microenvironment conducive for senescence even at younger age and have a significant impact on clinical outcomes (Rattanaprukskul et al. 2024). The DQ regimen was initially identified as a promising senotherapeutic agent through bioinformatic analyses, and subsequent research has validated its efficacy and safety in a range of diseases, also highlighting its potential to prolong life expectancy (Zhu et al. 2015; Xu et al. 2018; Chaib et al. 2022; Zhu et al. 2024). Using well-defined preclinical models and examining multiple markers of senescence, this study presents the first systematic evidence revealing that DQ can mitigate gingival senescence and thereby improve aging-associated periodontal bone loss.

Elevated levels of p16^INK4^ have been linked to cell-cycle arrest and decreased replication capacity of gingival keratinocytes after prolonged bacterial exposure in vitro as well as in gingival tissues of aged mice (Albuquerque-Souza et al. 2022; Albuquerque-Souza et al. 2024). These findings suggest that persistent stress from both internal and external sources can promote senescence characteristics and metabolic changes in the oral mucosa. We noted a reduction in p16 levels both in vivo and in vitro upon treatment with DQ, confirming the effectiveness of this drug regimen in alleviating senescence and promoting the maintenance of gingival tissue functionality. Our findings concurred with the results reported in other tissues when subjected to DQ administration. After radiation therapy, which is known to cause DNA damage and induce a senescent phenotype, mice treated with DQ exhibited decreased p16 levels, improved skin epithelial integrity, and fewer skin ulcerations (Wang et al. 2020). These findings indicate the potential application of senotherapeutics in this susceptible group. Similarly, in a kidney injury model, DQ application resulted in decreased p16 expression, a lower senescent cell burden, and enhanced proliferation of renal tubular epithelial cells (Li et al. 2021). Collectively, these findings corroborate the efficacy of DQ in mitigating cell-cycle arrest and fostering proliferative tissue repair in multiple tissues including oral mucosa.

Another hallmark of senescent cells is increased SA-β-galactosidase activity, indicative of enhanced lysosomal biogenesis (Gil 2023). Following administration of DQ, we noted diminished SA-β-galactosidase in the gingival tissues of aged mice as well as in gingival keratinocytes, which were subjected to prolonged bacterial exposure, suggesting improved lysosomal metabolism. Consistent with our findings, muscle and neural cells that were exposed to oxidative stress (H_2_O_2_) exhibited reduced SA-β-galactosidase upon DQ administration, resulting in improvements in physical and cognitive functions in vivo (Ota and Kodama 2022). Likewise, reduced SA-β-galactosidase and p16 levels were noted in adipocytes from obese patients following DQ treatment, supporting the efficacy of DQ across different cell types and conditions (Xu et al. 2018).

Due to the heterogeneity of senescent cells across tissues and diseases, characterization of a senescent cell can be better achieved through assessing multiple molecular signatures. Lipofuscin, a nondegradable lipoprotein that accumulates over time, is recognized as another reliable marker of senescence. An increase in both SA-β-galactosidase and lipofuscin levels is considered as a strong indicator of senescence and compromised lysosomal function and metabolic changes in response to cellular stress or damage (Georgakopoulou et al. 2013; Salmonowicz and Passos 2017). In this study, along with a significant reduction in SA-β-galactosidase levels, we also observed a marked decrease in lipofuscin accumulation within the gingival tissue of aged mice treated with DQ compared with vehicle-treated controls, aligning with the impact of DQ in other conditions such as metabolic dysfunction, intervertebral disc degeneration, and lung disease (Schafer et al. 2017; Novais et al. 2021; Islam et al. 2023). Furthermore, DQ treatment preserved nuclear membrane integrity in gingival keratinocytes, as shown by increased lamin-B1 levels. This result is consistent with findings in intestinal tissue of aged mice showing improved senescence phenotype after DQ treatment (Saccon et al. 2021). These findings mutually support the efficacy of the DQ drug regimen on senescence and cellular function, thereby improving tissue metabolism and health.

Periodontal tissues inflicted by disease exhibit increased levels of inflammatory mediators consistent with senescence secretome. Reducing the levels of these mediators through targeting senescence can foster regulated inflammation, resolution, and tissue homeostasis. Like the oral mucosa, the intestinal tract displays significant microbial diversity and acts as a physiological barrier against various stressors, which can increase susceptibility to senescence, particularly during aging. Similar to our observations, DQ has been shown to mitigate intestinal senescence by reducing senescent cells and SASP factors (Saccon et al. 2021). Notably, we also showed that DQ administration improves alveolar bone phenotype in aging mouse. Consistently, intermittent treatment with DQ over 6 wk led to increased bone volume and bone mineral density in the mandibular condyle in models of degenerative disorders of the temporomandibular joint associated with aging (Zhou et al. 2021). In addition, a recent study using a murine peri-implantitis model reported decreased senescence markers and osteoclasts and subsequently decreased peri-implant bone resorption and enhanced implant stability following DQ application (Yang et al. 2023). In another study, a short-term DQ treatment course diminished p16-positive senescent cells on the root surface during orthodontic tooth movement, resulting in reduced root resorption (Zhou et al. 2023). Together, growing evidence supports the efficacy of DQ interventions as a novel approach to sustain health, including the oral cavity.

In conclusion, our systematic approach has provided the first proof-of-concept evidence that DQ can mitigate gingival senescence and the senescence-associated secretome. This reduction may foster periodontal health by enhancing cellular metabolism and function while regulating inflammatory and healing processes, suggesting direct clinical implications. Foremost, the use of DQ is safe and effective, as determined by both preclinical and clinical studies. Long-term investigations have shown significant improvements in physical function and extended life span in DQ-treated mice, with no significant differences in the causes of death or morbidities compared with vehicle-treated groups, as determined through autopsies and complete metabolic and blood profiling (Xu et al. 2018; Novais et al. 2021). Clinical trials also report significantly improved outcomes with minimal or no side effects (Chaib et al. 2022; Nambiar et al. 2023). Finally, the efficacy of DQ can be sustained when applied as a “hit-and-run” approach, offering flexibility in intermittent or continuous administration (Chaib et al. 2022). Thus, senotherapy can be a promising novel approach to preserve periodontal health, particularly in susceptible populations either as a preventive or therapeutic approach. Future investigations will focus on translation of these promising findings into clinical practice through targeted strategies considering age, gender, and microbiome composition. The evidence of senescence in both gingival epithelial and connective tissues and the positive impact of senotherapy on aging alveolar bone also warrant investigating senescence-related events in other cells such as fibroblasts, macrophages, and osteocytes to gain further mechanistic insights.

## Author Contributions

K. Rattanaprukskul, contributed to conception and design, data acquisition, analysis, and interpretation, drafted and critically revised the manuscript; X.-Juan Xia, contributed to conception, data acquisition, analysis, and interpretation, critically revised the manuscript; M. Hysa, M. Jiang, contributed to acquisition, analysis, and interpretation, critically revised the manuscript; M. Hung, contributed to analysis and interpretation, critically revised the manuscript; S.F. Suslavich, contributed to analysis and interpretation, critically revised the manuscript; S.E. Sahingur, contributed to conception and design, data acquisition, analysis, and interpretation, drafted and critically revised the manuscript. All authors gave final approval and agreed to be accountable for all aspects of the work.

## Supplemental Material

sj-docx-1-jdr-10.1177_00220345241299789 – Supplemental material for Dasatinib and Quercetin Limit Gingival Senescence, Inflammation, and Bone Loss Supplemental material, sj-docx-1-jdr-10.1177_00220345241299789 for Dasatinib and Quercetin Limit Gingival Senescence, Inflammation, and Bone Loss by K. Rattanaprukskul, X.-J. Xia, M. Hysa, M. Jiang, M. Hung, S.F. Suslavich and S.E. Sahingur in Journal of Dental Research
