# Supplementary material for: Dasatinib and Quercetin Limit Gingival Senescence, Inflammation, and Bone Loss
Source: J Dent Res. 2025 Jan 10;104(4):419–27. doi: 10.1177/00220345241299789 (PMC11909784; doi:10.1177/00220345241299789)
Supplement: sj-docx-1-jdr-10.1177_00220345241299789 – Supplemental material for Dasatinib and Quercetin Limit Gingival Senescence, Inflammation, and Bone Loss [file sj-docx-1-jdr-10.1177_00220345241299789.docx]

**Title:** Dasatinib & quercetin limit gingival senescence, inflammation and bone loss

**Authors:** Kantapon Rattanaprukskul^1,2^; Xia-Juan Xia^1^; Min Jiang^1^; Megi Hysa^1^, Megan Hung^1^ Samuel Suslavich^1^and S. Esra Sahingur ^1#^

1. Department of Periodontics, School of Dental Medicine, University of Pennsylvania, Philadelphia, PA, USA.
2. Department of Periodontology, Faculty of Dentistry, Chulalongkorn University, Bangkok, Thailand

**APPENDIX**

1. **MATERIALS AND METHODS**
   1. ***IN VITRO* ASSAYS**
      1. **RNA isolation and RT-qPCR**

Total RNA was extracted using RNeasy Plus Mini Kit (Qiagen, #74136) and genomic DNA (gDNA) eliminator spin columns from the cells harvested following 24 hours of bacterial exposure as described (Mooney et al. 2021). The concentration and quality of RNA were measured by measurement of absorbance at 260/280 nm. Complementary DNA (cDNA) was synthesized using a High-Capacity cDNA reverse-transcription kit (Applied Biosystems, #4368814). Real-time quantitative polymerase chain reaction (RT-qPCR) was performed using specific primers for SYBR Green Master Mix (SABiosciences) in the StepOne Plus System (Applied Biosystems). The sequences of the primers are shown in **Appendix Table 1.** Relative mRNA levels were calculated by the ΔΔCT method using *GAPDH* as an endogenous control.

- - 1. **Western Blot**

Telomerase immortalized gingival keratinocytes (TIGKs) were harvested at day 6 and western blot was performed as described (Li et al. 2020). Briefly, the cells were rinsed with cold PBS, and subsequently lysed using RIPA lysis buffer (Cell Signaling) containing a protease inhibitor cocktail (Sigma-Aldrich, USA). The protein concentration was determined using Pierce^TM^ BCA protein assay kit (Thermo Fisher Scientific, MA, USA). Twenty micrograms of proteins of each sample were loaded into SDS-PAGE gels, separated by electrophoresis at 90V, 60mA, and then transferred to PVDF membranes (Millipore, MA, USA). Proteins were then detected by western blot, using mouse anti-p16^INK4a^ (Santa Cruz, SC-1661, USA), rabbit anti-Lamin-B1 (Proteintech), and mouse anti-GAPDH (Santa Cruz, sc-365062) at a concentration of 1: 500 as primary antibodies. HRP-linked anti-IgG (Bio-Rad) at a concentration of 1: 4,000 was used as a secondary antibody. Proteins were detected using SuperSignal™ West Pico PLUS Chemiluminescent Substrate (Thermo Fisher Scientific) and bands were captured on the FluorChem M System (BioTech, CA, USA). The intensity of the signal obtained for each protein was quantified by densitometry using Alpha View software and protein expression was calculated using GAPDH as endogenous control.

- - 1. **Immunofluorescence assay**

TIGKs were harvested at day 6 and immunofluorescence staining was performed as described (Albuquerque-Souza et al. 2023). The cells were fixed using 4% paraformaldehyde for 10 minutes at room temperature. Then cells were incubated with 0.3% TritonX- 100 (Sigma-Aldrich, #T8787) for 30 minutes at room temperature to permeabilize cells and block with 5% (w/v) bovine serum albumin for 1 hour to avoid unspecific binding of antibodies. The slide chambers were incubated with mouse anti-p16 (Santa Cruz, #sc-1661) and mouse anti-Lamin-B1 (Proteintech, #66095-1-ig) as primary antibodies diluted to 1:250 at 4°C overnight. Alexa-Fluor 488 goat anti-mouse IgG at 1:500 dilution was used as a secondary antibody. Cytoplasmic staining was performed using DyLight 554-phalloidin (F-actin) (Cell Signaling). Coverslips were mounted with a drop of the Vectashield^®^ antifade mounting media with DAPI (Vector Laboratories) and images were taken using a confocal Nikon laser microscope (Nikon Instruments) at 630x magnification or Leica DM6B microscope (Leica) at 40X magnification.

- - 1. **SA-β-galactosidase staining**

SA-β-galactosidase is one of the most commonly used markers of cellular senescence. International Cell Senescence Association recommends SA-β-galactosidase as the initial marker for assessing senescence in combination with p16 and other selected markers and SASPs (Gil 2023)*.* SA-β-galactosidase staining was performed on the cells harvested at day 6 with SA-β-galactosidase staining kit (Cell Signaling Technology #9860) (Albuquerque-Souza et al. 2023). Images were obtained using a digital inverted-phase microscope (Olympus CK40) at a 20x magnification. After staining, TIGKs were lysed using RIPA buffer (Sigma-Aldrich), and SA-β-galactosidase activity was measured using the microplate reader (Thermo Fisher Scientific Multiskan MCC) at OD 600 nm (Albuquerque-Souza et al. 2023; Ewald et al. 2009).

- - 1. **Cell viability assay**

Cell viability was determined by using the Cell Counting Kit-8 (APExBIO) assay. TIGKs were seeded into 96-well plates at 5 × 10^3^ cells/well and cultured overnight. Then, the cells were cultured with and without D (10 nM), Q (1 μM), and the combination of DQ for 1,3,5 and 7 days. CCK-8 reagent was added (10 μl/well) and the plate was incubated for another 2 hours. The absorbance at 450 nm was measured using a microplate reader (Thermo Fisher Scientific Multiskan MCC).

- 1. ***IN VIVO* STUDIES**
     1. **Histology of mouse gingival tissues**

The gingival tissues surrounding maxillary molars, excluding buccal mucosa and palatal tissues, were excised, fixed in 4% paraformaldehyde and dehydrated using a 30% sucrose solution before being embedded in OCT for cryosection (Mooney et al. 2021). The samples were then cut at a thickness of 5 µm at -20°C using a cryostat. For SA-β-galactosidase staining, the frozen sections were dried at 37°C for 20-30 minutes. Subsequently, the samples were stained with SA-β-galactosidase staining solution (Cell Signaling, #9860) overnight at 37°C in a dry incubator, following the manufacturer’s instructions. Frozen sections stained with SA-β-galactosidase were quantified using ImageJ software to measure the SA-β-galactosidase-positive area. The percentage of positive areas was calculated by dividing the stained area by the total tissue area and multiplying by 100 (Crowe and Yue 2019; Jannone et al. 2020). Lipofuscin deposition in the gingival tissues was detected using Sudan Black B (SBB) solution following established protocols (Georgakopoulou et al. 2013). Lipofuscin staining was considered positive when perinuclear and cytoplasmic aggregates of blue-black granules were visible within the cells. Lipofuscin-positive cells were manually quantified in each field using a 40x magnification, and a minimum of three randomly selected regions/fields were evaluated for quantification. Immunofluorescence staining for p16 was performed on gingival tissue sections as described previously (Rattanaprukskul et al. 2024). Briefly, the frozen sections were fixed with 4% paraformaldehyde at room temperature. Non-specific binding sites were blocked by incubation with with 5% (w/v) goat serum in TPBS in a humidity chamber at room temperature for an hour. Then, the tissue samples were incubated with anti-p16 mouse monoclonal antibody (Santa Cruz Biotech, SC1661, 1:50) overnight at 4 °C. The following day, the sections were rinsed with PBS and incubated with secondary antibody goat anti-mouse Alexa-Fluor 488 (Invitrogen, A11001, 1:400) for 2 hours. Then, the sections were mounted on slides with mouthing media (Vectashield) and DAPI (Vector Laboratories, CA, USA, H-1800). The images were taken using Leica DM6B microscope (Leica, Germany) at 40X magnification. Three randomly selected images per sample were analyzed. The relative fluorescence intensity and the percentage of colocalization were calculated using ImageJ Fiji software. Regions of interest (ROI) primarily in the connective tissue area, including a portion of the epithelial layer from the gingival margin.

- - 1. **Alveolar bone loss assessments**

Alveolar bone levels were determined using a Nikon microscope (Nikon Instrument Inc., SMZ800) with 40X objective and NIS-Elements software (Nikon Instrument Inc.) following established protocols (Kim et al. 2015). The distance from the cementoenamel junction (CEJ) to the alveolar bone crest (ABC) was averaged from six sites on the maxillary molars as follows: the disto-palatal and palatal cusps of the first molar, the mesio-palatal cusp, palatal groove, and disto-palatal cusp of the second molar, and the palatal groove of the third molar.

**
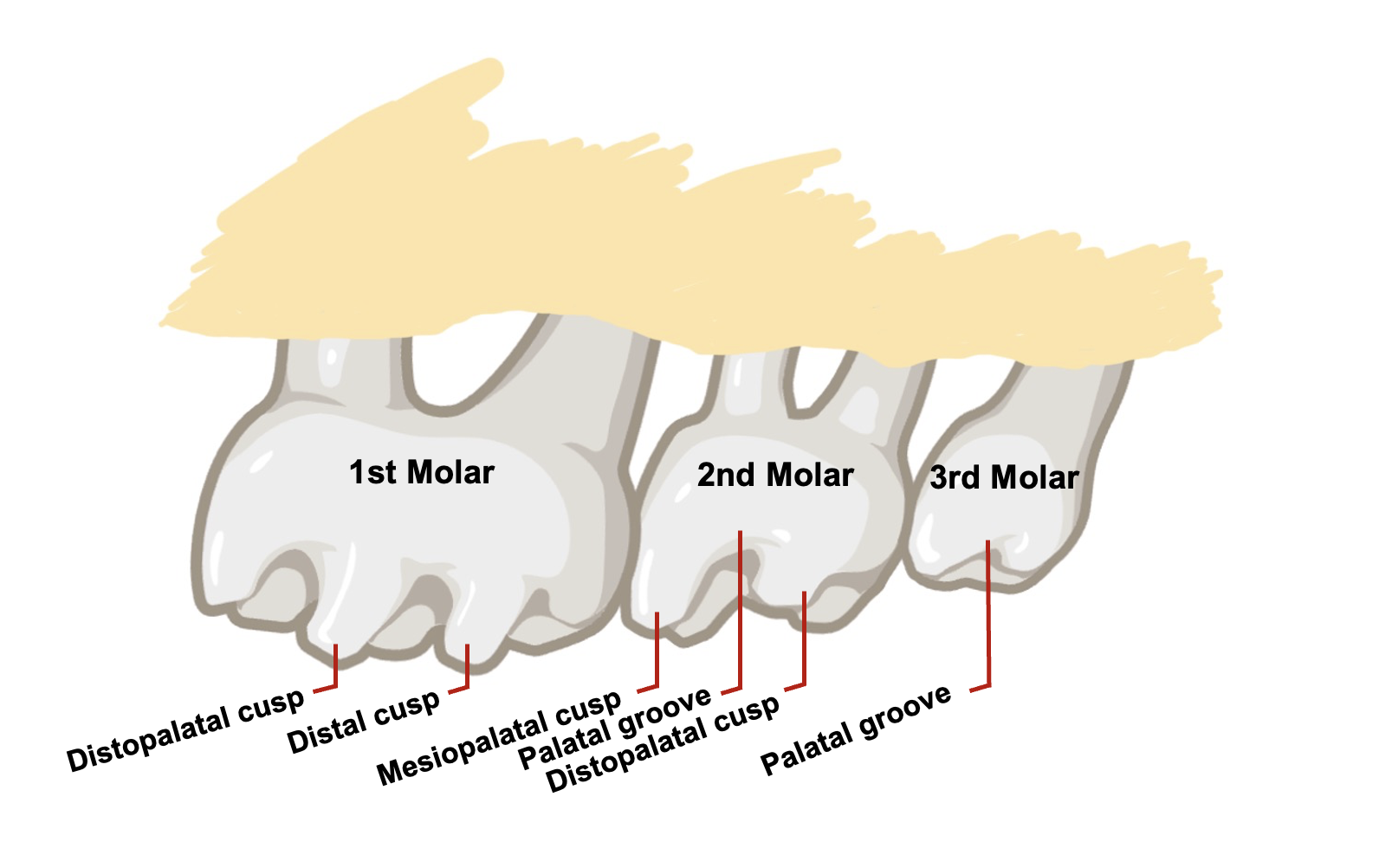
**

**Appendix Figure 1.** Schematics depicting the sites where measurements of the CEJ-ABC distance were conducted.

**
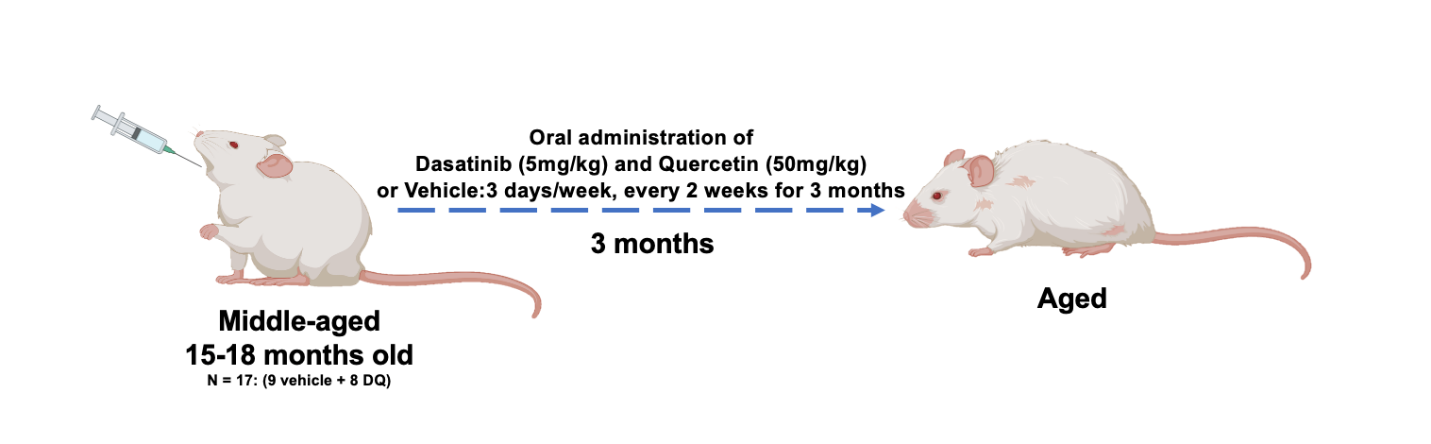
**

**Appendix Figure 2. Schematic model of DQ treatment in murine naturally progressing periodontal bone loss model.** Middle-age BALB/c mice were orally administered D (5 mg/kg) and Q (50 mg/kg) for 3 consecutive days per week, every 2 weeks during the 3-month period of treatment. After the treatment, mice were euthanized, and periodontal tissues were examined.

**
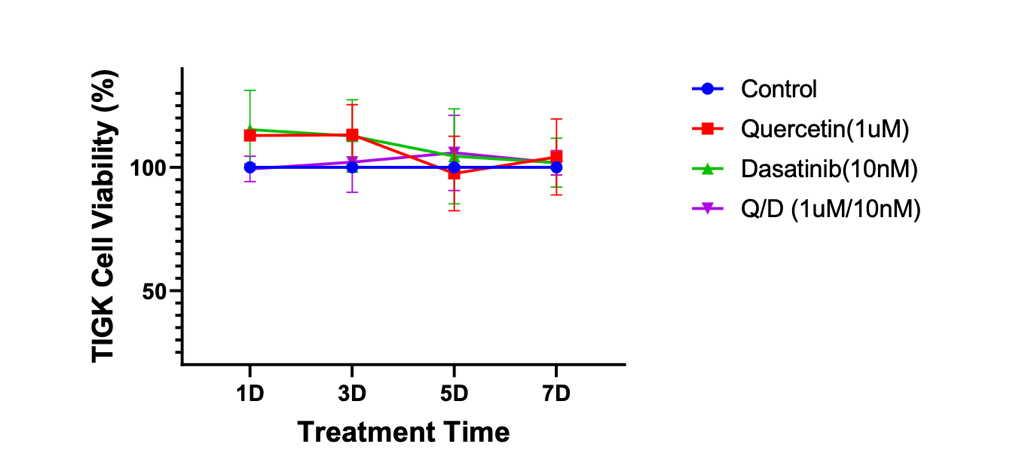
**

**Appendix Figure 3.** Cell viability of gingival keratinocytes treated with 1 µM Q, 10 nM D, and DQ were measured by CCK-8 colorimetric assays.


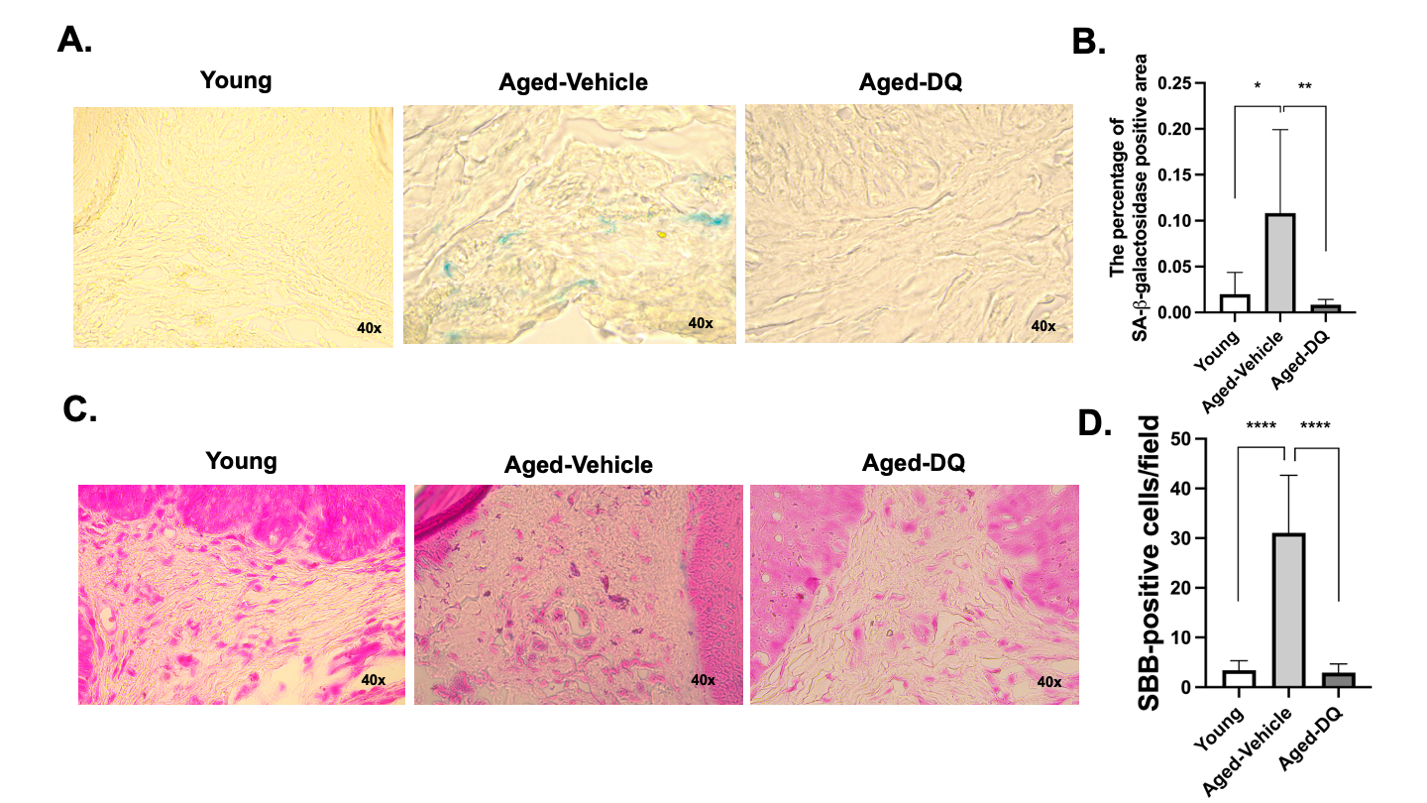


**Appendix Figure 4. DQ reduces gingival senescence.** Balb/c mice (15-16 months old) were treated with either vehicle (n=9) or DQ (n=8) for 3 consecutive days every two weeks over a 3-month period. Balb/c mice (9-10 weeks old) served as younger controls (n=6). Senescence markers included SA-β-galactosidase (A, B) and lipofuscin deposition (C, D).

**Appendix Table 1.** The sequences of the primers for *in vitro*.

| **HUMANS** | **Sequences (5’-3’)** |
| --- | --- |
| **RT-qPCR** |  |
| *GAPDH* | Forward: CAA TGA CCC CTT CAT TGA CC |
|  | Reverse: TTG ATT TTG GAG GGA TCT CG |
| *IL-8* | Forward: ACT GAG AGT GAT TGA GAG TGG AC |
|  | Reverse: AAC CCT CTG CAC CCA GTT TTC |
| *MMP-1* | Forward: CTC TGG AGT AAT GTC ACA CCT CT |
|  | Reverse: TGT TGG TCC ACC TTT CAT CTT C |
| *MMP-3* | Forward: CTG GAC TCC GAC ACT CTG GA |
|  | Reverse: CAG GAA AGG TTC TGA AGT GAC C |

**Appendix Table 2.** The sequences of the primers for *in vivo*.

| **MICE** | **Sequences (5’-3’)** |
| --- | --- |
| **RT-qPCR** |  |
| *Gapgh* | Forward: AGG TCG GTG TGA ACG GAT TTG |
|  | Reverse: GGG GTC GTT GAT GGC AAC A |
| *P16* | Forward: ATC TGG AGC AGC ATG GAG TC |
|  | Reverse: GGG GTA CGA CCG AAA GAG TT |
| *Il-1β* | Forward: GTT TCT GCT TTC ACC ACT CCA |
|  | Reverse: GAG TCC AAT TTA CTC CAG GTC AG |
| *Il-8* | Forward: TCG AGA CCA TTT ACT GCA ACA G |
|  | Reverse: CAT TGC CGG TGG AAA TTC CTT |
| *Tnf-α* | Forward: CTG AAC TTC GGG GTG ATC GG |
|  | Reverse: GGC TTG TCA CTC GAA TTT TGA GA |
| *Mmp-13* | Forward: ACT TCT ACC CAT TTG ATG GAC CTT |
|  | Reverse: AAG CTC ATG GGC AGC AAC A |
| *Mmp-3* | Forward: ACA TGG AGA CTT TGT CCC TTT TG |
|  | Reverse: TTG GCT GAG TGG TAG AGT CCC |

**Appendix Table 3.** Sex differences in the expression of senescence markers in mice.

|  | **Sex** | **Vehicle**  Mean (SD) | **DQ**  Mean (SD) | ***p*-Value** |
| --- | --- | --- | --- | --- |
| SA-β-galactosidase | F | 0.13 (0.08) | 0.01(0.01) | 0.0082** |
|  | M | 0.03(0.02) | 0.01(0.00) | 0.3388 (ns) |
| Lipofuscin | F | 26.19 (6.18) | 2.55 (1.21) | <0.0001**** |
|  | M | 48.17 (4.97) | 4.13 (2.13) | 0.0147* |
| P16 (Fluorescence) | F | 1.03 (0.10) | 0.53 (0.11) | 0.0014** |
|  | M | 1.00 (0.00) | 0.54 (0.00) | Too small of sample size |
| P16 (mRNA) | F | 1.04 (0.37) | 0.36 (0.12) | 0.0058** |
|  | M | 1.20 (0.36) | 0.92 (0.06) | >0.9999 (ns) |
| IL-1β | F | 1.08 (0.34) | 0.32 (0.13) | 0.0025** |
|  | M | 0.72 (0.00) | 0.28 (0.02) | Too small of sample size |
| IL-8 | F | 1.06 (0.76) | 0.08 (0.04) | 0.0393* |
|  | M | 2.05 (0.00) | 0.23 (0.04) | Too small of sample size |
| TNF-α | F | 1.27 (0.92) | 0.49 (0.20) | 0.1109 (ns) |
|  | M | 0.97 (0.20) | 0.68 (0.12) | 0.6667 (ns) |
| MMP-13 | F | 1.06 (0.45) | 0.56 (0.14) | 0.0177* |
|  | M | 0.89 (0.00) | 0.62 (0.03) | Too small of sample size |
| MMP-3 | F | 1.15 (0.96) | 0.35 (0.23) | 0.0303* |
|  | M | 0.57 (0.00) | 0.51 (0.06) | Too small of sample size |

**References**

Albuquerque-Souza E, Shelling B, Jiang M, Xia XJ, Rattanaprukskul K, Sahingur SE. 2023. Fusobacterium nucleatum triggers senescence phenotype in gingival epithelial cells. Mol Oral Microbiol.

Crowe AR, Yue W. 2019. Semi-quantitative determination of protein expression using immunohistochemistry staining and analysis: An integrated protocol. Bio Protoc. 9(24).

Ewald JA, Peters N, Desotelle JA, Hoffmann FM, Jarrard DF. 2009. A high-throughput method to identify novel senescence-inducing compounds. J Biomol Screen. 14(7):853-858.

Georgakopoulou EA, Tsimaratou K, Evangelou K, Fernandez Marcos PJ, Zoumpourlis V, Trougakos IP, Kletsas D, Bartek J, Serrano M, Gorgoulis VG. 2013. Specific lipofuscin staining as a novel biomarker to detect replicative and stress-induced senescence. A method applicable in cryo-preserved and archival tissues. Aging (Albany NY). 5(1):37-50.

Gil J. 2023. The challenge of identifying senescent cells. Nat Cell Biol. 25(11):1554-1556.

Jannone G, Rozzi M, Najimi M, Decottignies A, Sokal EM. 2020. An optimized protocol for histochemical detection of senescence-associated beta-galactosidase activity in cryopreserved liver tissue. J Histochem Cytochem. 68(4):269-278.

Li Y, Mooney EC, Xia XJ, Gupta N, Sahingur SE. 2020. A20 restricts inflammatory response and desensitizes gingival keratinocytes to apoptosis. Front Immunol. 11:365.

Mooney EC, Holden SE, Xia XJ, Li Y, Jiang M, Banson CN, Zhu B, Sahingur SE. 2021. Quercetin preserves oral cavity health by mitigating inflammation and microbial dysbiosis. Front Immunol. 12:774273.

Rattanaprukskul K, Xia XJ, Jiang M, Albuquerque-Souza E, Bandyopadhyay D, Sahingur SE. 2024. Molecular signatures of senescence in periodontitis: Clinical insights. J Dent Res. 103(8):800-808.
